# Evaluation of the quality of life before and after rehabilitation with dental implants: a pilot study

**DOI:** 10.1080/07853890.2021.1897345

**Published:** 2021-09-28

**Authors:** Tiago Pereira Troles, Catarina Godinho, Luís Proença, Pedro Rodrigues, José João Mendes

**Affiliations:** a Instituto Universitário Egas Moniz (IUEM), Egas Moniz Cooperativa de Ensino Superior, Caparica, Portugal; b Centro de Investigação Interdisciplinar Egas Moniz (CiiEM), Egas Moniz Cooperativa de Ensino Superior, Caparica, Portugal; c Egas Moniz Dental Clinic (EMDC), Egas Moniz Cooperativa de Ensino Superior, Caparica, Portugal

## Abstract

**Introduction:**

The World Health Organisation (WHO) defines quality of life as “an individual's perception of their position (…) and in relation to their goals, expectations, standards and concerns” [1]. It is also known that dental implants restore function, aesthetics and phonetics, factors that, in turn, influence quality of life [2]. The Oral Health Impact Profile (OHIP) is used to assess oral health self-perception [3]. This pilot study was aimed to evaluate and compare the patient’s quality of life, before and after oral rehabilitation with dental implants.

**Materials and methods:**

After study approval by the Ethics Committee of Egas Moniz, CRL, and following the informed consent, a validated Portuguese version of the OHIP, in its short-form (OHIP-14), was applied in order to allow the profiling of socio-demographic and oral health characteristics of the patients, as well as to measure their self-perception. Data analysis was performed by using descriptive and inferential statistics methodologies. In the later, a significance level of 5% was established.

**Results:**

A total of 23 patients, from the Egas Moniz Dental Clinic (EMDC), designated for rehabilitation with dental implants, with a mean age of 51.9 (±15.5) years, were included in the study. The sample was characterised, predominantly, by females, married, with an active status and a higher level of education. From those, 14 patients were evaluated before and after rehabilitation with dental implants. The overall OHIP-14 mean score changed from 18.8 (±11.0) to 2.5 (±2.1). Before rehabilitation the highest OHIP-14 subscale score was the one related to the “psychological discomfort” domain. An improvement of the quality of life was observed not only for the overall OHIP-14 index score (median decreased from 16.5 to 2.0) but also for each of the OHIP domains: “functional limitation” (2.5–1.0, *p* = .012), “physical pain” (3.5–0.0, *p* = .001), “psychological discomfort” (5.0–0.5, *p* = .002), “physical disability” (3.0–0.0, *p* = .003), “psychological disability” (2.5–0.0, *p* = .002), “social disability” (1.0–0.0, *p* = .006) and “handicap” (1.0–0.0, *p* = .007).

**Discussion and conclusions:**

There were significant improvements on the patient’s quality of life through rehabilitation with dental implants, as noted by the decrease in measured self-perception scores before and after rehabilitation. The changes were identified for all of the seven OHIP-14 domain scores indicating an overall improvement in the patient’s quality of life.
